# Role of C-Peptide in the Regulation of Microvascular Blood Flow

**DOI:** 10.1155/2008/176245

**Published:** 2008-07-28

**Authors:** T. Forst, T. Kunt, B. Wilhelm, M. M. Weber, A. Pfützner

**Affiliations:** ^1^Medical Department, Institute for Clinical Research and Development, 55116 Mainz, Germany; ^2^Department of Endocrinolgy and Metabolism, Johannes Gutenberg University, 55131 Mainz, Germany; ^3^Diabetes Centre, 13187 Berlin, Germany

## Abstract

During the recent years, the role of C-peptide, released from the pancreatic beta cell, in regulating microvascular blood flow, has received increasing attention. In type 1 diabetic patients, intravenous application of C-peptide in physiological concentrations was shown to increase microvascular blood flow, and to improve microvascular endothelial function and the endothelial release of NO. C-peptide was shown to impact microvascular blood flow by several interactive pathways, like stimulating Na^+^K^+^ATPase or the endothelial release of NO. There is increasing evidence, that in patients with declining beta cell function, the lack of C-peptide secretion might play a putative role in the development of microvascular blood flow abnormalities, which go beyond the effects of declining insulin secretion or increased blood glucose levels.

## 1. INTRODUCTION

Patients with diabetes mellitus type 1 present with an extensive risk for microvascular complications like retinopathy, nephropathy, and peripheral neuropathy. Although hyperglycemia is recognized as a major driver in the development of these diabetic complications, the precise mechanism, whereby diabetes precipitates these complications, is not fully understood. Furthermore, also in type 1 diabetic patients with good metabolic control, the risk for the development of microvascular complications is reduced but still not abolished. In the DCCT trial, type 1 diabetic patients with sustained C-peptide secretion showed a significant smaller risk for microvascular complications compared with those patients totally lacking C-peptide secretion from the beta cell [1]. In this study, even modest beta cell activity was associated with a decrease in the incidence of microvascular complications.

Regulation of vascular tone is a dynamic process, regulated by a complex interaction of several balancing and counterbalancing forces. The kinetics of postprandial insulin, C-peptide, and blood glucose levels was shown to interact in the regulation of microvascular blood flow in several tissues like the skin or the heart [2, 3]. Although it is not possible to separate the beneficial effects of residual C-peptide secretion from those of residual insulin secretion, there is increasing evidence that C-peptide might play a putative role in the physiology of microvascular blood flow regulation.

In type 1 diabetes mellitus, numerous functional alterations in blood flow could be observed early after beta cell dysfunction has emerged [4, 5]. Early type 1 diabetes is characterised by increased microvascular blood flow, increased shear stress, and tangential pressure on the microvascular endothelium. In addition, increased leukocyte-endothelial adhesion [6], increased blood viscosity [7, 8], and changes in the haemodynamic properties of red blood cells [9, 10] further affect microvascular blood flow. These early functional disturbances proceed structural alterations in the vessel wall, including basement membrane thickening as well as arteriolar hyalinosis [11].

The role of vascular endothelium for micro- and macrovascular blood flow regulation has been extensively investigated within the last decade [12, 13]. The endothelial cells coat the internal lumen of the vessels and serve as an interface between circulating blood cells and the vascular smooth muscle cell. In addition to serve as a physical barrier between the blood and the underlying smooth muscle cells, the endothelial cell facilitates a complex array of signalling between the vessel wall and the enclosed blood compartment. There are several transmitters released from endothelial cells like nitric oxide (NO), endothelin 1, prostaglandins, thrombin, substance P, bradykinin, serotonin, and others which impact the vascular tone [14, 15].

**Figure 1:**
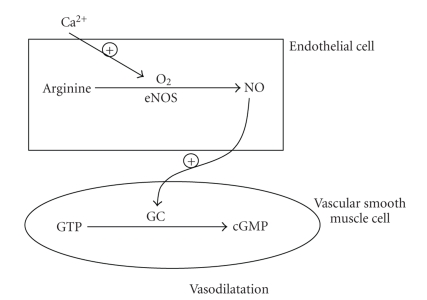
Mechanism of endothelial nitric oxide synthesis with stimulation of guanylcyclase in the vascular smooth muscle cell and subsequent vasorelaxation.

Nitric oxide was identified as the primary vasodilator released from the endothelium [16]. As shown in Figure 1, NO elicits vasodilatation through stimulation of endothelial NO-synthase (eNOS), increasing the endothelial release of NO and subsequent activation of the guanylcyclase in the vascular smooth muscle cell [12, 17–19].

**Figure 2:**
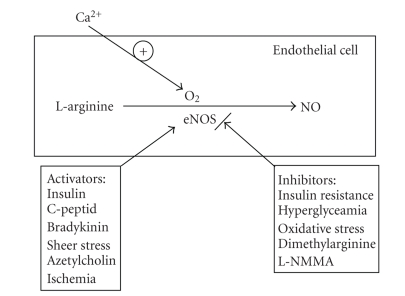
Substrates known to activate or reduce the endothelial nitric oxide synthase system.

As shown in Figure 2, the activity of eNOS could be stimulated or suppressed by several signaling molecules, known to be altered in patients with diabetes mellitus. Reduced levels of circulating NO contribute to vascular injury by facilitating platelet-vascular wall interaction, increasing the adhesion of circulating monocytes to the endothelial surface, and stimulation of vascular smooth muscle proliferation [20]. Impaired endothelial function and a reduction in endothelial NO release are early features of type 1 diabetes and thought to be principal causes of morbidity and mortality in these patients.

## 2. EFFECTS OF C-PEPTIDE ON NITRIC OXIDE (NO)

**Figure 3:**
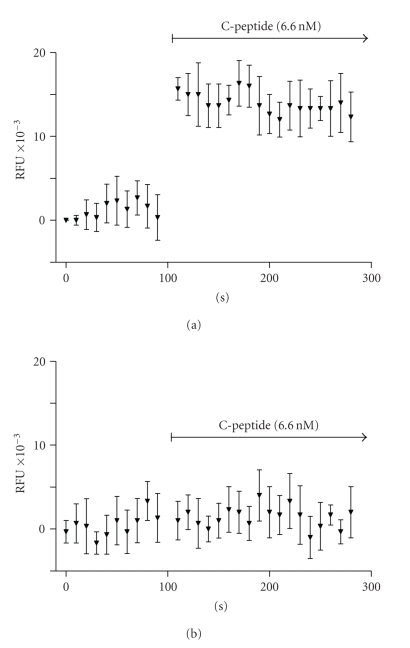
C-peptide induced calcium influx into endothelial cells. Effect of C-peptide on the Ca2+ signal in endothelial cells loaded with Fluo-3. Addition of C-peptide to bovine aortic endothelial cells yielded in a significant increase in fluorescence (above), which was not found in calcium free medium (b).

C-peptide was shown to significantly enhance the release of NO from bovine aortic endothelial cells (BAECs) in a dose-dependent manner [21, 22]. The release of NO in this study was dose dependent and already obtained within the physiological range of 1–6 nM. C-peptide increased the intracellular Ca^2+^ concentration in BAEC (see Figure 3). Since the endothelial eNOS is a Ca^2+^/calmodulin-regulated enzyme [23], both the C-peptide-stimulated Ca^2+^ signal and the NO release were abolished in Ca^2+^-free medium. Therefore, the peptide is likely to stimulate eNOS activity by facilitating an influx of Ca^2+^ into BAEC.

The NO release from BAEC declined from 2–30 minutes of incubation, indicating a desensitization of the potential receptor, or the subsequent signalling cascade, which has been also demonstrated for other peptide signals for endothelial NO release [24]. In conclusion, C-peptide is able to stimulate an influx of Ca^2+^ into endothelial cell, thereby activating the Ca^2+^-sensitive endothelial NO synthetase and stimulating the release of NO from the endothelial cell.

In a study by Kitamura et al., C-peptide was shown to stimulate NO production by enhancing the mitogen-activated protein-kinase dependent transcription of endothelial nitric synthase in aortic endothelial cells of Wistar rats [25]. In this study, it was shown that C-peptide increases NO release from aortic endothelial cells by enhancing eNOS expression through an ERK-dependent transcriptional pathway.

## 3. EFFECTS OF C-PEPTIDE ON ERYTHROCYTE Na^+^K^+^ATPASE

Na^+^K^+^ATPase activity has been found to be attenuated in various cell types under diabetic conditions [26–28]. It has also been shown that hyperglycemia inhibits Na^+^K^+^ATPase activity by an endothelium dependent mechanism [29]. Na^+^K^+^-ATPase is a plasma membrane-associated protein complex, expressed in most eukaryotic cells. It couples the energy released from the intracellular hydrolysis of ATP to the transport of cellular ions, a major pathway for the controlled translocation of sodium and potassium ions across the cell membrane. Therefore, Na^+^K^+^-ATPase controls directly or indirectly many essential cellular functions, for example, cell volume, free calcium concentrations, and membrane potential [30]. Although there are tissue specific differences in the regulations of Na^+^K^+^-ATPase activity, hyperglycemia and diabetes are predominantly characterized by a decrease in ouabain-sensitive Na^+^K^+^-ATPase activity. This would result in an increase in intracellular calcium concentration and an increased vascular tone, promoting the development of vascular complications in diabetes mellitus. Na^+^K^+^ATPase activity is involved in vascular regulation based on a complex interaction between Na^+^K^+^-pump-activity and an endothelium dependent increase of NO [31, 32]. On the other hand, NO and cyclic-GMP have been shown to increase vascular Na^+^K^+^ATPase activity, with subsequent vasorelaxation [33, 34].

In order to hypothesize the potential mechanism of C-peptide activity, previous studies concerning Na^+^K^+^ATPase activity in erythrocytes and renal tubular cells are of considerable interest [9, 35, 36]. Ohtomo et al. were able to show that the attenuated activity of Na^+^-K^+^-ATPase activity in renal tubular segments of diabetic rats is restored by C-peptide. On the other hand, an attenuation of Na^+^-K^+^-ATPase activity has been demonstrated to correlate with decreased erythrocyte deformability in type 1 diabetic patients [9].

In a recent study, erythrocyte Na^+^K^+^ATPase activity was found to be reduced in type 1 diabetic patients, while in type 2 diabetic patients a wide range of individual Na^+^K^+^ATPase activities was observed, presenting some patients with very low Na^+^K^+^ATPase activity and others with a normal Na^+^K^+^ATPase activity. It appeared that erythrocyte Na^+^K^+^ATPase activity was significantly lower in those type 2 diabetic patients treated with insulin compared with those on oral treatment. Also in the former, Na^+^K^+^ATPase activity was comparable to those in type 1 diabetic patients.

In an in vitro study by Djemli-Shiplolye et al., incubation of erythrocytes from type 1 diabetic patients with C-peptide normalized erythrocyte Na^+^K^+^ATPase activity [37]. In another study, intravenous infusion of C-peptide was found to improve erythrocyte Na^+^K^+^ATPase activity in type 1 diabetic patients [38].

## 4. EFFECT OF C-PEPTIDE ON RED CELL DEFORMABILITY

Blood flow in larger vessels is determined by the vessel diameter, blood viscosity, and vessel length according to the law of Hagen-Pouiseuille. In the capillary bed, especially if the diameter of the vessel is below the diameter of the erythrocytes, blood flow is predominantly determined by the viscosity and deformability of the erythrocytes. Thus, reduced erythrocyte deformability will reduce blood flow if the capillary diameter and blood pressure remain constant [39].

Several studies demonstrated that factors such as decreased erythrocyte deformability, increased erythrocyte aggregation, and increased cell membrane rigidity contribute to alterations in microvascular blood flow in patients with diabetes mellitus [7, 9, 10, 40–44].

Concerning the possible mechanism of reduced erythrocyte deformability, it is noteworthy that Na^+^-K^+^-ATPase activity has been shown to be attenuated in several cell types, including erythrocytes in diabetic patients [9, 35, 36], and that it may be restored not only by insulin but C-peptide as well [35].

**Figure 4:**
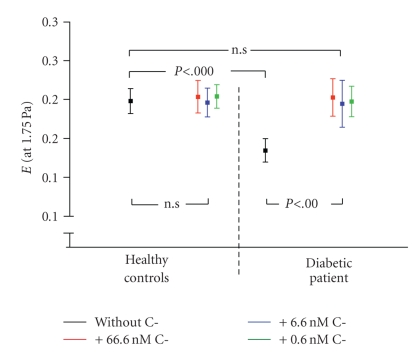
*Representative analysis of erythrocyte deformability at 1.75 Pa*. This graph shows the alterations of elongation index E at a shear stress of 1.75 Pa, which is frequently achieved in vivo. C-peptide did not modify the deformability of erythrocytes obtained from healthy controls, whereas the deformability of diabetic erythrocytes was restored to normal levels after administration of different concentrations of the peptide. Statistical analysis was performed by Student's *t* test.

The deformability of erythrocytes in type 1 diabetic patients was found to be reduced compared to healthy controls [22]. Both groups were matched concerning their glucose levels in order to exclude a glucotoxic effect. Deformability was tested under physiological (0.3 to 10 Pa) and supraphysiological (>10 Pa) shear stress rates by means of laser diffractoscopy. Incubation of erythrocytes from healthy controls and type 1 diabetic patients with different concentrations of C-peptide restored erythrocyte deformability in type 1 diabetic patients but was without any effect in erythrocytes of nondiabetic controls (see Figure 4).

It is speculative to discuss the underlying mechanism based upon these results, but impaired Na^+^K^+^ATPase activity may contribute to the decrease in erythrocyte deformability by increasing the intracellular sodium concentration with subsequent intracellular accumulation of free calcium ions due to competition in transmembranous exchange [45]. These abnormalities in calcium homeostasis are known to enhance spectrin dimer-dimer interaction and spectrin-protein 4.1-actin interaction [46, 47]. The latter is being promoted by adducin, a membrane-skeleton-associated calmodulin-binding protein [48].

Pretreatment of erythrocytes from type 1 diabetic patients with ouabain, EDTA, or pertussis toxin completely abolished C-peptide effects on erythrocyte deformability as shown in more detail in the paper of Hach et al. in the same issue of Experimental Diabetes Research.

## 5. EFFECTS OF C-PEPTIDE ON MICROVASCULAR BLOOD FLOW

In several studies, C-peptide was shown to affect microvascular blood flow and to improve nerve or renal function in animal models of type 1 diabetes and in humans with type 1 diabetes mellitus [49–52]. In a study by Lindstrom et al., C-peptide supplementation was shown to increase microvascular blood flow and to enhance the recruitment of capillaries in isolated kidneys of the rat [53]. In another study, the effect of C-peptides was investigated in skeletal muscle arterioles isolated from rat cremaster muscles [54]. In this study, C-peptide evoked a concentration independent arteriolar dilatation in a range between 0.3 and 1000 ng/mL. Addition of insulin at low concentrations, which had no vascular effect by its own, enhanced the vascular effect of C-peptide, indicating a permissive role of both pancreatic peptides in the regulation of microvascular blood flow. Inhibition of the nitric oxide synthase by LNMA completely abolished the vasodilating response to C-peptide, further stressing the role of NO in the transmission of C-peptide vascular effects.

In a study done by Ido et al., beneficial effects of C-peptide supplementation could be documented in several vascular beds in diabetic rats [55]. In their study, biosynthetic human C-peptide was given subcutaneously twice daily for 5 weeks in control rats and streptozotocin-induced diabetic rats. Highly supraphysiological peak plasma C-peptide levels between 9 and 10 nM were reached. C-peptide markedly reduced the diabetes induced increase in blood flow in the anterior uvea, retina, and sciatic nerve. In addition, C-peptide prevented increased ^125^I-labeled albumin permeation in retina, nerve, and in the aorta. The effect on microvascular blood flow was accompanied by an increase in caudal motor nerve conduction velocity. No effect of C-peptide, neither on microvascular blood flow nor on motor nerve conduction velocity, could be observed in the healthy control rats. Cotter et al. observed the vascular effects of C-peptide on sciatic endoneurial blood flow in streptozotocin diabetic rats at physiological C-peptide concentrations [49]. In their study, C-peptide supplementation revealed an improvement in endoneurial blood flow and vascular conductance by 57 and 66%, respectively. The increase in endoneurial blood flow was accompanied by an improvement in motor nerve conduction velocity by 62% and in sensory nerve conduction velocity by 78%. Again, treatment with L-NNA abolished the effect of C-peptide on endoneurial blood flow and nerve conduction velocity.

In an investigation by Johanssen et al., the effect of C-peptides on skeletal muscle blood flow was observed in type 1 diabetic patients and in healthy controls during exercise [56]. In the type 1 diabetic subjects, blood flow and capillary diffusion capacity of the exercising forearm at baseline were approximately 30% lower compared to the healthy control subjects. Intravenous administration of C-peptide increased forearm blood flow by 27% and capillary diffusion capacity by 52% to levels similar to those observed in the healthy controls. No significant changes in blood flow could be observed in healthy controls receiving C-peptide or in diabetic patients receiving placebo infusion. In accordance with the observed improvements in muscle blood flow, forearm oxygen and glucose uptake increased markedly after C-peptide administration in type 1 diabetic patients.

**Figure 5:**
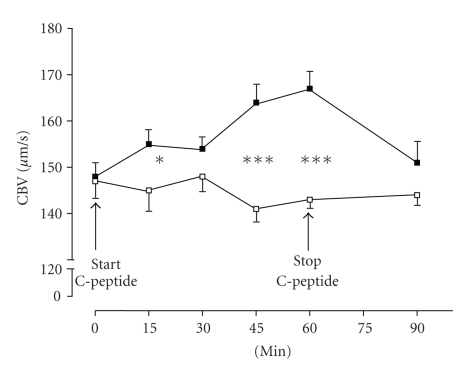
Skin capillary during intravenous application of C-peptide (8 pmol/kg/min) in type 1 diabetic patients (■) and non-diabetic controls (□) (mean ± SEM; * = *P* < .05; *** = *P* < .001).

Skin blood flow is affected early after the diagnosis of diabetes mellitus [57–59]. The skin capillary circulation is functionally situated in parallel to the arteriovenous shunts and is thought to have the primary function of tissue nutrition. It has been estimated that 80–90% of total skin blood flow passes through thermoregulatory arteriovenous shunts and does not enter the nutritive part of the capillary bed [60–62]. While total skin perfusion is increased in diabetes mellitus, nutritional capillary skin blood flow was shown to be reduced in diabetic patients [60, 61, 63]. As shown in Figure 5, short-term infusion of C-peptide in type 1 diabetic patients was found to redistribute microvascular blood flow from the subpapillary thermoregulatory blood flow into the nutritive capillary bed [64]. At baseline, nutritive capillary blood flow was significantly lower in type 1 diabetic patients compared with the control group. C-peptide supplementation in type 1 diabetic patients increased capillary blood flow to a level comparable to that observed in the healthy control group. Thirty minutes after the termination of the C-peptide infusion, capillary blood flow had declined to a level not different from baseline levels. No such effect of C-peptide application on microvascular skin blood flow could be observed in nondiabetic subjects. A linear relationship was found between plasma C-peptide levels and the capillary blood flow velocity (*r* = 0.401; *P* < .0001).

Fernqvist-Forbes et al. studied the effect of C-peptide on flow-mediated vasodilatation (FMD) in type 1 diabetic patients [65]. In addition, the arterial dilatation to glyceryl trinitrate, which is an endothelium independent marker of vascular smooth muscle function, was investigated. When compared with the healthy control group, the type 1 diabetic patients revealed a lower FMD. Following C-peptide administration, blood flow in the brachial artery increased by approximately 35%, and FMD was significantly improved. No effect of C-peptide could be observed on the microvascular response to glyceryl trinitrate, which further confirm the endothelium dependent pathway of C-peptide.

**Figure 6:**
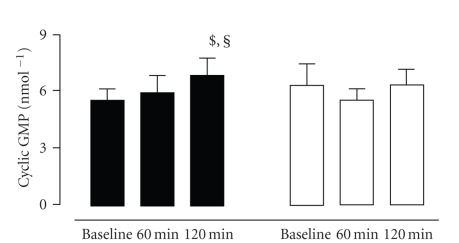
Cyclic guanyl monophosphate (cGMP) at baseline and after 60 and 120 minutes of C-peptide (■) or placebo (□) (mean ± SEM; $ = *P* < .05 versus baseline; § = *P* < .05 versus 60 minutes).

As shown in Figure 1, acetylcholine elicits vasodilatation through a stimulation of endothelial NO-synthase (eNOS) with an increase in the endothelial release of NO and a subsequent stimulation of the guanylcyclase in the vascular smooth muscle cell. In a recent study, the effect of intravenous C-peptide infusion on the acetylcholine induced increase in microvascular blood flow was investigated in type 1 diabetic patients [38]. Skin microvascular response was measured by laser Doppler fluxmetry, and acetylcholine was applied to the dorsum of the foot using the technique of iontophoresis. The microvascular response to acetylcholine increased by 133% during short-term infusion of C-peptide, which was accompanied by a significant increase in plasma cyclic GMP levels (see Figure 6).

In contrast, in a study of Polska et al., no effect of C-peptide supplementation in type 1 diabetic patients could be observed on retinal blood flow [66].

Therefore, it could be postulated that C-peptide affects microvascular blood flow in a tissue specific manner.

## 6. CONCLUSIONS

**Figure 7:**
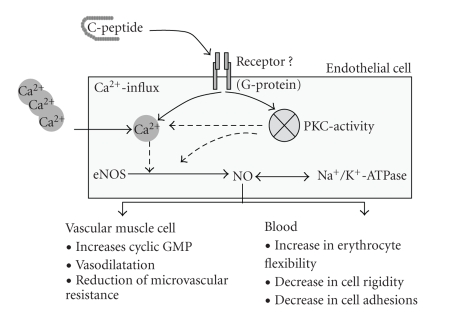
Schematic presentation of the molecular mechanism of C-peptide activity on endothelial cells and microvascular blood flow.

Insulin depletion in type 1 diabetic patients results in hyperglycaemia and the development of vascular complications of diabetes mellitus. Treatment of type 1 diabetes mellitus with insulin replacement is an effective tool for addressing glucose metabolism, but it seems conceivable that the loss of C-peptide secretion from pancreatic beta cells might contribute to the vascular complications in patients with diabetes mellitus type 1. As shown in this review, recent studies showed that C-peptide is biologically active by modulating endothelial function and microvascular blood flow. The underlying mechanisms involve the activation of endothelial nitric oxide synthase and the activation of Na^+^K^+^ATPase, which was shown to be calcium-dependent and ouabain sensitive. The postulated mechanism by which C-peptide interact with microvascular blood flow is illustrated in Figure 7.

Since the vascular effects of C-peptide could not be confirmed in all tissues, it seems conceivable that there are tissue specific differences in the mode of C-peptides vascular activities. Instead a specific binding of C-peptide to the cell membrane could be demonstrated [67], no specific receptor for C-peptide could be isolated neither from endothelial cells nor from other cell systems. Therefore, there is still a substantial need for the further investigation of the molecular effects of C-peptide on cellular level.

Nevertheless, the improvement in erythrocyte flexibility and microvascular blood flow after C-peptide supplementation in type 1 diabetic patients encourages the claim for further prospective interventional trials to establish the clinical relevance for C-peptide supplementation in type 1 diabetic patients.

## References

[1] Steffes MW, Sibley S, Jackson M, Thomas W (2003). *β*-cell function and the development of diabetes-related complications in the diabetes control and 
complications trial. *Diabetes Care*.

[2] Scognamiglio R, Negut C, De Kreutzenberg SV, Tiengo A, Avogaro A (2005). Postprandial myocardial perfusion in healthy subjects and in type 2 diabetic patients. *Circulation*.

[3] Forst T, Forst S, Strunk K (2005). Impact of insulin on microvascular blood flow and endothelial cell function in the postprandial state in patients with type 1 diabetes. *Journal of Diabetes and Its Complications*.

[4] Tibiriçá E, Rodrigues E, Cobas RA, Gomes MB (2007). Endothelial function in patients with type 1 diabetes evaluated by skin capillary recruitment. *Microvascular Research*.

[5] Forst T, Pfützner A, Kunt T (1998). Skin microcirculation in patients with type I diabetes with and without neuropathy after 
neurovascular stimulation. *Clinical Science*.

[6] Kunt T, Forst T, Harzer O (1998). The influence of advanced glycation endproducts (AGE) on the expression of human endothelial adhesion molecules. *Experimental and Clinical Endocrinology and Diabetes*.

[7] Ernst E, Matrai A (1986). Altered red and white blood cell rheology in type II diabetes. *Diabetes*.

[8] Barnes AJ, Locke P, Scudder PR, Dormandy TL, Dormandy JA, Slack J (1977). Is hyperviscosity a treatable component of diabetic microcirculatory disease?. *The Lancet*.

[9] Finotti P, Palatini P (1986). Reduction of erythrocyte (Na^+^-K^+^)ATPase activity in type 1 (insulin-dependent) diabetic subjects and its activation by homologous plasma. *Diabetologia*.

[10] McMillan DE, Utterback NG, La Puma J (1978). Reduced erythrocyte deformability in diabetes. *Diabetes*.

[11] Tooke JE (1995). Microvascular function in human diabetes: a physiological perspective. *Diabetes*.

[12] Calles-Escandon J, Cipolla M (2001). Diabetes and endothelial dysfunction: a clinical perspective. *Endocrine Reviews*.

[13] Kuo L, Davis MJ, Chilian WM (1988). Myogenic activity in isolated subepicardial and subendocardial coronary arterioles. *American Journal of Physiology*.

[14] Colwell JA, Lopes-Virella MF (1988). A review of the development of large-vessel disease in diabetes mellitus. *American Journal of Medicine*.

[15] Furchgott RF, Zawadzki JV (1980). The obligatory role of endothelial cells in the relaxation of arterial smooth muscle by acetylcholine. *Nature*.

[16] Palmer RMJ, Ferrige AG, Moncada S (1987). Nitric oxide release accounts for the biological activity of endothelium-derived relaxing factor. *Nature*.

[17] Morris SJ, Shore AC, Tooke JE (1995). Responses of the skin microcirculation to acetylcholine and sodium nitroprusside in patients with NIDDM. *Diabetologia*.

[18] Pieper GM, Siebeneich W, Moore-Milton G, Roza AM (1997). Reversal by L-arginine of a dysfunctional arginine/nitric oxide pathway in the endothelium of the 
genetic diabetic BB rat. *Diabetologia*.

[19] McNally PG, Watt PAC, Rimmer T, Burden AC, Hearnshaw JR, Thurston H (1994). Impaired contraction and endothelium-dependent relaxation in isolated resistance vessels from patients with insulin-dependent diabetes mellitus. *Clinical Science*.

[20] Johnstone MT, Creager SJ, Scales KM, Cusco JA, Lee BK, Creager MA (1993). Impaired endothelium-dependent vasodilation in patients with insulin-dependent diabetes mellitus. *Circulation*.

[21] Wallerath T, Kunt T, Forst T (2003). Stimulation of endothelial nitric oxide synthase by proinsulin C-peptide. *Nitric Oxide*.

[22] Kunt T, Schneider S, Pfützner A (1999). The effect of human proinsulin C-peptide on erythrocyte deformability in patients with type I diabetes mellitus. *Diabetologia*.

[23] Förstermann U, Pollock JS, Schmidt HH, Heller M, Murad F (1991). Calmodulin-dependent endothelium-derived relaxing factor/nitric oxide synthase activity is present in the particulate and cytosolic fractions of bovine aortic endothelial cells. *Proceedings of the National Academy of Sciences of the United States of America*.

[24] Förstermann U, Mugge A, Alheid U, Haverich A, Frolich JC (1988). Selective attenuation of endothelium-mediated vasodilation in atherosclerotic human coronary arteries. *Circulation Research*.

[25] Kitamura T, Kimura K, Jung BD (2002). Proinsulin C-peptide activates cAMP response element-binding proteins through the p38 mitogen-activated protein kinase pathway in mouse lung capillary endothelial cells. *Biochemical Journal*.

[26] Wald H, Scherzer P, Rasch R, Popovtzer MM (1993). Renal tubular Na^+^-K^+^-ATPase in diabetes mellitus: relationship to metabolic abnormality. *American Journal of Physiology*.

[27] Gerbi A, Barbey O, Raccah D (1997). Alteration of Na,K-ATPase isoenzymes in diabetic cardiomyopathy: 
effect of dietary supplementation with fish oil (n-3 fatty acids) in rats. *Diabetologia*.

[28] Vague P, Dufayet D, Coste T, Moriscot C, Jannot MF, Raccah D (1997). Association of diabetic neuropathy with Na/K ATPase gene polymorphism. *Diabetologia*.

[29] Simmons DA, Kern EF, Winegrad AI, Martin DB (1986). Basal phosphatidylinositol turnover controls aortic Na^+^/K^+^ ATPase activity. *The Journal of Clinical Investigation*.

[30] Rose AM, Valdes R (1994). Understanding the sodium pump and its relevance to disease. *Clinical Chemistry*.

[31] Rapoport RM, Schwartz K, Murad F (1985). Effects of Na^+^,K^+^-pump inhibitors and membrane depolarizing agents on acetylcholine-induced endothelium-dependent relaxation 
and cyclic GMP accumulation in rat aorta. *European Journal of Pharmacology*.

[32] Tack CJJ, Lutterman JA, Vervoort G, Thien T, Smits P (1996). Activation of the sodium-potassium pump contributes to insulin-induced vasodilation in humans. *Hypertension*.

[33] Gupta S, McArthur C, Grady C, Ruderman NB (1994). Stimulation of vascular Na^+^-K^+^-ATPase activity by nitric oxide: a cGMP-independent effect. *American Journal of Physiology*.

[34] Rand VE, Garland CJ (1992). Endothelium-dependent relaxation to acetylcholine in the rabbit basilar artery: importance of membrane hyperpolarization. *British Journal of Pharmacology*.

[35] Ohtomo Y, Aperia A, Sahlgren B, Johansson B-L, Wahren J (1996). C-peptide stimulates rat renal tubular Na^+^,K^+^-ATPase activity in synergism with neuropeptide Y. *Diabetologia*.

[36] Ohtomo Y, Bergman T, Johansson B-L, Jörnvall H, Wahren J (1998). Differential effects of proinsulin C-peptide fragments on Na^+^,K^+^-ATPase activity of renal tubule segments. *Diabetologia*.

[37] Djemli-Shipkolye A, Gallice P, Coste T (2000). The effects ex vivo and in vitro of insulin and C-peptide on Na/K adenosine triphosphatase activity in red blood cell membranes of type 1 diabetic patients. *Metabolism*.

[38] Forst T, Dufayet De La Tour D, Kunt T (2000). Effects of proinsulin C-peptide on nitric oxide, microvascular blood flow and erythrocyte Na^+^,K^+^-ATPase activity in diabetes mellitus type I. *Clinical Science*.

[39] Chien S (1987). Red cell deformability and its relevance to blood flow. *Annual Review of Physiology*.

[40] Bareford D, Jennings PE, Stone PC, Baar S, Barnett AH, Stuart J (1986). Effects of hyperglycaemia and sorbitol accumulation on erythrocyte deformability in diabetes mellitus. *Journal of Clinical Pathology*.

[41] Chimori K, Miyazaki S, Kosaka J, Sakanaka A, Yasuda Y, Miura K (1986). Increased sodium influx into erythrocytes in diabetes mellitus and hypertension. *Clinical and Experimental Hypertension*.

[42] Cohen NS, Ekholm JE, Luthra MG, Hanahan DJ (1976). Biochemical characterization of density separated human erythrocytes. *Biochimica et Biophysica Acta*.

[43] Baba Y, Kai M, Kamada T, Setoyama S, Otsuji S (1979). Higher levels of erythrocyte membrane microviscosity in diabetes. *Diabetes*.

[44] Schmid-Schönbein H, Volger E (1976). Red-cell aggregation and red-cell deformability in diabetes. *Diabetes*.

[45] Mazzanti L, Rabini RA, Faloia E, Fumelli P, Bertoil E, De Pirro R (1990). Altered cellular Ca^2+^ and Na^+^ transport in diabetes mellitus. *Diabetes*.

[46] Takakuwa Y, Mohandas N (1988). Modulation of erythrocyte membrane material properties by Ca^2+^ and calmodulin. Implications for their role in regulation of skeletal protein interactions. *The Journal of Clinical Investigation*.

[47] Schischmanoff PO, Winardi R, Discher DE (1995). Defining of the minimal domain of protein 4.1 involved in spectrin-actin binding. *Journal of Biological Chemistry*.

[48] Gardner K, Bennett V (1986). A new erythrocyte membrane-associated protein with calmodulin binding activity. 
Identification and purification. *Journal of Biological Chemistry*.

[49] Cotter MA, Ekberg K, Wahren J, Cameron NE (2003). Effects of proinsulin C-peptide in experimental diabetic neuropathy: vascular actions and modulation by nitric oxide synthase inhibition. *Diabetes*.

[50] Kamiya H, Zhang W, Ekberg K, Wahren J, Sima AAF (2006). C-peptide reverses nociceptive neuropathy in type 1 diabetes. *Diabetes*.

[51] Johansson B-L, Borg K, Fernqvist-Forbes E, Kernell A, Odergren T, Wahren J (2000). Beneficial effects of C-peptide on incipient nephropathy and neuropathy in patients with 
type 1 diabetes mellitus. *Diabetic Medicine*.

[52] Ekberg K, Juntti-Berggren L, Norrby A (2005). C-peptide improves sensory nerve function in type 1 diabetes and neuropathy. *Diabetologia*.

[53] Lindström K, Johansson C, Johnsson E, Haraldsson B (1996). Acute effects of C-peptide on the microvasculature of isolated perfused skeletal muscles and kidneys in rat. *Acta Physiologica Scandinavica*.

[54] Jensen ME, Messina EJ (1999). C-peptide induces a concentration-dependent dilation of skeletal muscle arterioles only in presence of insulin. *American Journal of Physiology*.

[55] Ido Y, Vindigni A, Chang K (1997). Prevention of vascular and neural dysfunction in diabetic rats by C-peptide. *Science*.

[56] Johansson B-L, Linde B, Wahren J (1992). Effects of C-peptide on blood flow, capillary diffusion capacity and glucose utilization in the exercising forearm of type 1 (insulin-dependent) diabetic patients. *Diabetologia*.

[57] Tooke JE, Lins PE, Ostergren J, Fagrell B (1985). Skin microvascular autoregulatory responses in type I diabetes: the influence of duration and control. *International Journal of Microcirculation, Clinical and Experimental*.

[58] Ewald U, Tuvemo T, Rooth G (1981). Early reduction of vascular reactivity in diabetic children detected by transcutaneous oxygen electrode. *The Lancet*.

[59] Flynn MD, Tooke JE (1992). Aetiology of diabetic foot ulceration: a role for the microcirculation?. *Diabetic Medicine*.

[60] Jörneskog G, Brismar K, Fagrell B (1995). Skin capillary circulation is more impaired in the toes of diabetic than non-diabetic patients with peripheral vascular disease. *Diabetic Medicine*.

[61] Jörneskog G, Brismar K, Fagrell B (1995). Skin capillary circulation severely impaired in toes of patients with IDDM, with and without late diabetic 
complications. *Diabetologia*.

[62] Boulton AJM, Scarpello JHB, Ward JD (1982). Venous oxygenation in the diabetic neuropathic foot: evidence of arteriovenous shunting?. *Diabetologia*.

[63] Jörneskog G, Ostergren J, Tyden G, Bolinder J, Fagrell B (1990). Does combined kidney and pancreas transplantation reverse functional diabetic microangiopathy?. *Transplant International*.

[64] Forst T, Kunt T, Pohlmann T (1998). Biological activity of C-peptide on the skin microcirculation in patients with insulin-dependent diabetes mellitus. *The Journal of Clinical Investigation*.

[65] Fernqvist-Forbes E, Johansson B-L, Eriksson MJ (2001). Effects of C-peptide on forearm blood flow and brachial artery dilatation in patients with type 1 diabetes mellitus. *Acta Physiologica Scandinavica*.

[66] Polska E, Kolodjaschna J, Berisha F (2006). C-peptide does not affect ocular blood flow in patients with type 1 diabetes. *Diabetes Care*.

[67] Rigler R, Pramanik A, Jonasson P (1999). Specific binding of proinsulin C-peptide to human cell membranes. *Proceedings of the National Academy of Sciences of the United States of America*.

